# Antibody-mediated Prevention of *Fusarium* Mycotoxins in the Field

**DOI:** 10.3390/ijms9101915

**Published:** 2008-10-09

**Authors:** Zu-Quan Hu, He-Ping Li, Jing-Bo Zhang, Elena Glinka, Yu-Cai Liao

**Affiliations:** 1 Molecular Biotechnology Laboratory of Triticeae Crops, Huazhong Agricultural University, Wuhan 430070, P.R. China. E-Mails: huzuquan@webmail.hzau.edu.cn (Z. H.); hepingli@mail.hzau.edu.cn (H. L.); jingbozhang@webmail.hzau.edu.cn (J. Z.); 2 College of Plant Science and Technology, Huazhong Agricultural University, Wuhan 430070, P.R. China; 3 Laboratory of Structural Biochemistry, Shemyakin and Ovchinnikov Institute of Bioorganic Chemistry, Russian Academy of Sciences, 117997 Moscow, Russia. E-Mails: em_glinka@mail.ru (E. G.)

**Keywords:** Antibody fusion, *Fusarium* mycotoxins, single-chain variable fragment

## Abstract

*Fusarium* mycotoxins directly accumulated in grains during the infection of wheat and other cereal crops by *Fusarium* head blight (FHB) pathogens are detrimental to humans and domesticated animals. Prevention of the mycotoxins via the development of FHB-resistant varieties has been a challenge due to the scarcity of natural resistance against FHB pathogens. Various antibodies specific to *Fusarium* fungi and mycotoxins are widely used in immunoassays and antibody-mediated resistance *in planta* against *Fusarium* pathogens has been demonstrated. Antibodies fused to antifungal proteins have been shown to confer a very significantly enhanced *Fusarium* resistance in transgenic plants. Thus, antibody fusions hold great promise as an effective tool for the prevention of mycotoxin contaminations in cereal grains. This review highlights the utilization of protective antibodies derived from phage display to increase endogenous resistance of wheat to FHB pathogens and consequently to reduce mycotoxins in field. The role played by *Fusarium*-specific antibody in the resistance is also discussed.

## 1. Introduction

Mycotoxins are secondary metabolites that are produced by many different genera of fungi including *Aspergillus*, *Fusarium* and *Penicillium* [1, 2]. Infection by mycotoxin-producing fungi such as *Fusarium* head blight (FHB) pathogens takes place mainly during the flowering period of small grain cereal crops in field and consequently mycotoxins produced during the infection directly accumulate in grains, and thus enter food/feed chains. *Fusarium* mycotoxins are among the main fungal mycotoxin contaminations in food and livestock in China, and some human diseases, such as Kashi-Neck diseases and esophageal cancer, have been epidemiologically associated with consumption of trichothecence mycotoxins [3]. To prevent mycotoxin contaminations in cereal grains, reduction of the pathogen infection in field by endogenous expression of resistance genes is a key step. However, no germplasm exists that provides effective innate resistance to *Fusarium* mycotoxin-producing pathogens under high disease pressure [4, 5] and the development of resistant cereal varieties with suitable agronomic traits has been a challenge with conventional strategies [6, 7]. Current protective measures rely heavily on the chemical control of pathogens, with severe and undesirable environmental consequences. Alternative approaches are therefore required to protect plants against FHB pathogens and to reduce mycotoxin production [8–11].

Antibodies, or immunoglobulins, are defense molecules synthesized by all vertebrates in response to the presence of a foreign substance, called an antigen. They display defined specificity and affinity for the antigens that elicited their synthesis. Antibodies recognize and bind substance-specific antigens and thus help to eliminate substances from the body. Various antibodies specific for *Fusarium* mycotoxins and pathogens have been generated. Fungus-specific antibodies have been shown to reduce fungal growth *in vitro* [12] and to prevent infection of the host plants by the fungal pathogen [13]. Monoclonal and recombinant antibodies have been expressed in plants. Plant-derived antibodies have been developed for the protection of plants against pathogens [14–19] and immunomodulation [20, 21] in addition to their therapeutic applications [22]. Expression in plants of antibodies specific for mycotoxin-producing pathogens can restrict the spreading of the pathogens in field and eventually reduce mycotoxin-production load. This review highlights some recent advances of antibody-based prevention of *Fusarium* mycotoxins in filed, with emphasis on application of antibody fusion proteins in cereal crops.

## 2. Antibodies specific for *Fusarium* mycotoxins and mycotoxin-producing fungi

With the invention of hybridoma technology [23], monoclonal antibodies with high binding specificity to *Fusarium* mycotoxins and mycotoxin-producing fungi are isolated and widely used in immunoassays [24–28]. However, monoclonal antibodies are expensive to produce and maintain because specialized cell cultures and costly low-temperature storage facilities are required. In addition they carry two heavy chains and two light chains, and thus it is difficult to genetically manipulate them to construct fusion proteins with other partners. Rapid progress in molecular immunology, combined with the polymerase chain reaction, has made it possible to clone the antibody binding domain (Fv fragment) and express the polypeptide chains in bacteria, yeast, mammalian cells and plant cells either as pure antibodies or as fusion proteins comprising antibodies genetically linked to other peptides [29, 30]. By advanced technologies such as phage display, antibody fragments specific for particular antigens can be isolated *in vitro* from libraries containing diverse repertoires of antibodies V-genes, which bypasses hybridoma technology altogether and generates single-chain antibodies with specificity and affinity similar to monoclonal antibodies. This is based on the fact that the difference in antigen-binding specificities between antibodies lies entirely within their variable regions that are directly involved in the interaction with antigens. Therefore, it is necessary only to isolate genes encoding for the variable domains, which can then be jointed to constant regions by recombinant techniques [31, 32]. These technical advances in recombinant antibody production have been applied widely to research in the plant science and biotechnology, and play an important role in the reduction of *Fusarium* mycotoxin-producing pathogens in cereals.

**Figure 1.**
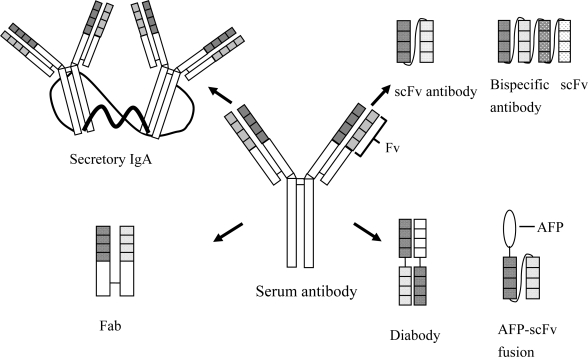
Different antibody forms expressed in transgenic plants

In phage display, each phage displays a single antibody fragment comprising the variable regions of the heavy and light chains that form the Fv domains of natural antibodies, which is called a single-chain variable fragment (scFv). The scFv gene contained in the phagemid encodes for the scFv antibody displayed on the surface of a phage particle, and thus phage display directly links phenotype (scFv antibody) and genotype (scFv gene) [32]. Specific scFv antibodies are selected by panning of phage displayed antibodies in solid or solution phase. For the solid phase panning, immunotubes were coated with antigens and after washing bound pages are eluted for subsequent infection of *E. coli*. Phages isolated from the bacteria are used for the next round of panning on the antigens. Solution phase panning can be carried out by incubating the phage library with biotinylated antigens and capturing the complex on streptavidin coated paramagnetic beads. Eluted phages are subjected to bacterial infection as described for the solid phase panning. A total of three rounds of panning is usually performed. Phage displayed antibodies are screened for binding to the antigens by enzyme-linked immunosorbent assay (ELISA). After *Bst*NI fingerprinting of ELISA-positive clones, the nucleotide sequences encoding for scFv antibodies can be determined. scFv antibody genes can be manipulated genetically with ease for the construction and expression of new recombinant proteins, such as AFP (antifungal peptide)-scFv fusions described in Figure 1 and in the succeeding sections. Numerous recombinant antibodies have been isolated from human [33], mice [34], chicken [35], sheep [36] and other animals [37, 38]. In addition, isolated scFv genes can be subjected further to mutation, and the mutants with more desirable characteristics then can be selected [39, 40]. Phage display antibodies can be used in the same range of applications as their hybridoma counterparts. From immunocompetent phage display libraries constructed with spleenic RNAs from chicken, Peschen et al. [16] have isolated several single-chain antibodies specific to antigens displayed on the *Fusarium* cell surface. Western blot analyses and immunofluorescence labelings confirm that these antibodies react strongly with cell wall-bound proteins and bind to the surface components of *F. asiaticum*, a predominant FHB species in China [41]. One of highly specific phage display scFv antibodies has been used for the protection of plants against *Fusarium* pathogens and for the construction of antibody fusion proteins [16, 42].

## 3. Antibody expression in plants

Antibody expression in plants was pioneered by Hiatt and colleagues [43] and Düring and colleagues [44]. These researchers demonstrated that plants can express and assemble functionally active antibodies with virtually identical specificity and affinity as monoclonal antibodies produced by hybridoma cell lines. Since then, various forms of antibodies, including secretory IgA antibodies [45], full-size serum antibodies [46], Fab fragments [47], single-chain variable fragments (scFvs) [14, 17, 48], biscFvs [49], diabodies [50] or antibody fusions comprising an antibody and an antifungal peptide (AFP) [16, 42], have been functionally expressed in a diverge range of plants (Figure 1) by using the same pathway as mammalian cells for the assembly of light and heavy chains involving similar signal peptides and successful folding [51]. Leader peptides derived from both heavy and light chains of a mouse monoclonal antibody efficiently target the proteins into Golgi apparatus and then into apoplast in plant cells [19, 46]. Moreover, plants have significant advantages over other expression systems and can carry out many of the post-translational modifications required for optimal biological activity of the antibodies [52].

Expression of antibodies and their proper assembly and transport in plants have resulted in an increasing awareness that this strategy could be utilized for neutralizing and blocking plant pathogens, and thus for generating resistant plants. Cytosolic expression of a single-chain antibody against artichoke mottled crinkle virus in transgenic tobacco has been shown to reduce viral infection and delay the progression of disease symptoms [17]. Also, secretion into the apoplast of a full-size antibody recognizing intact tobacco mosaic virus particles was shown to reduce the number of local necrotic lesions in transgenic tobacco [46]. Recently the expression of a *Fusarium*-specific single-chain antibody *in planta* has been shown to confer *Fusarium* resistance in transgenic *Arabidopsis* plants similar to that of expressing an antifungal protein [16]. These results indicate that protective antibodies specific to pathogens have a great potential for conferring pathogen resistance in plants.

Antibodies can be expressed either transiently in plant leaves or stably in transgenic plants, depending on applications required. Both expression systems in plants have been well established and widely exploited to produce antibodies for basic research and the pharmaceutical, agricultural and biotechnological industries [15, 18, 21, 22, 53, 54]. Transient expression in plants is a fast expression system for structural and functional characterization of antibodies without generation of transgenic plants [54]. It is usually used for verifying that the alien gene product, for instance, animal-derived antibodies, is functional and stable prior to generating transgenic plants. Vacuum infiltration is utilized for the delivery of recombinant *Agrobacterium* cells into intact leaf tissue, where the antibody gene is expressed. The leaf tissue is used only for transient protein production and no selection to identify transformed cells is required. The transferred T-DNA does not get integrated into plant chromosome but is present in the nucleus, where it is transcribed, and this leads to transient expression of the antibody. Expression levels observed in the transient assay are very consistent with those obtained with transgenic approach. We routinely use this system to test expression of antibodies or genes of interest, and to study their functions before moving to generating transgenic plants [29, 55].

Stable expression in plants involves generating transgenic plants where the antibody gene is stably integrated into the plant genome and expressed throughout different generations. With the advances of biotechnology, many crops have been successfully transformed via *Agrobacterium tumefaciens* or projectile bombardment, including wheat, barley, maize, oat and rice that are frequently infected by FHB pathogens. Transgenic cereal crops without any genes or sequences encoding for antibiotics or herbicides can be generated [56, 57]. Different innate protein sorting and targeting sequences that plant cells normally use have been used for expression of recombinant antibodies in transgenics. Significant increase of antibody expression level has been achieved when antibodies are targeted to the secretory pathway instead of the cytosol [19]. Endoplasmic reticulum (ER) retention can give 10- to 100-fold higher level of antibody expression and ER can be used as cellular storage apparatus for large scale production of antibodies or important proteins [58]. Antibody expressed in plant seeds is stable during storage at room temperature for at least one and half years without significant loss of functional antibody content [59]. Thus stably expressed antibodies in cereal grains can be active for the prevention of *Fusarium* mycotoxin-producing pathogens during storage for a long period of time.

## 4. Antibody-mediated prevention of *Fusarium* mycotoxins in field and postulated mechanisms

*Fusarium* mycotoxins are produced by *Fusarium* species that cause an economically devastating disease, called *Fusarium* head blight, on wheat and other small grain cereal crops worldwide [5]. In China the first instance of FHB was reported in 1936 and since then FHB epidemics have become more severe and frequent in the middle and lower regions of the Yangtze River, and in Heilongjiang province in northeastern China [3]. FHB has re-emerged as a serious threat to agriculture in Europe and North America since the middle of 1990s [5, 60, 61], causing losses of billions of US dollars. Over recent decades considerable effort has been made in China and other countries to breed FHB resistant cultivars [7, 62–64] and no commercial cultivars has been bred with available natural FHB resistance germplasms. Antifungal proteins (AFPs), such as chitinases, have been expressed in plants to confer FHB disease resistance [8, 55, 65]. In most cases, however, the expression of individual AFPs only delays the appearance of disease symptoms and does not provide effective control of the disease.

To evaluate the potential of antibody-mediated FHB resistance in plants, Peschen *et al*. [16] generate specific antibodies against *Fusarium asiaticum*, the predominant *Fusarium* species in wheat FHB epidemic regions in China [41, 66] and a producer of the mycotoxin deoxynivalenol [67]. This strategy is based on the use of *Fusarium*-specific antibodies that target AFPs to the site of infection by binding to surface components of the invading fungus in plants in order to directly interfere with fungal growth and development. Thus, *Fusarium* cell wall-bound proteins are selected as the target for the protective antibodies, since these proteins are displayed on the pathogen surface during infection. A chicken-derived phage display scFv antibody with a high-affinity is identified that reacts strongly with cell wall-bound proteins from *Fusarium* pathogens [16].

This antibody confers a significantly enhanced resistance in transgenic *Arabidopsis thaliana* plants upon infection with *Fusarium* pathogens 14 days post inoculation (dpi). More importantly, when the coding sequence of this antibody is genetically fused to any of three AFPs, the resulting AFP-scFv fusion proteins display strong inhibitory activity on the growth of *Fusarium* spp. *in vitro*, whereas under the same conditions no inhibitory activity is observed in the mixtures of antibodies and AFPs that are separately expressed. In transgenic *Arabidopsis thaliana* plants 21 dpi, a very significantly enhanced resistance to the pathogens is seen only for plants expressing these scFv-AFP fusions but not for the plants expressing the fusions containing a non-*Fusarium*-specific scFv antibody and the same AFPs. Therefore, the presence of a *Fusarium*-specific antibody is essential for the inhibitory activity and the enhanced resistance. Furthermore, no difference between the transgenic plants and nontransgenic controls after inoculation with a non-*Fusarium* fungal species, *Sclerotinia sclerotiorum* indicates that this resistance is *Fusarium*-specific [16]. These results indicated that antibody-mediated resistance is pathogen-specific and thus the antibody fusions expressed in plants are not harmful to other microorganisms in the environment, implying that the antibody-based approach is an environmentally friendly strategy for protection of plants against pathogens.

Recently, further analyses of transgenic wheat plants reveal highly significantly enhanced resistance for plants expressing an AFP-scFv fusion after single-floret injection and spraying inoculation with *F. asiaticum* [42]. Up to 86 and 79% of reduction in spikelet infection are achieved in T2 and T3 transgenic wheat plants, respectively, 21 dpi with single-floret injection, compared with that of the nontransgenic wheat controls. These results indicated that the antibody fusion protein confers the resistance of wheat plants to *Fusarium* spreading (i.e. type II resistance). Also at 21 dpi after spray inoculation, T3 transgenic wheat plants show a significant disease reduction of up to 41% in area under the disease progress curve (AUDPC) compared with the nontransgenic wheat, suggesting an enhanced resistance of wheat plants to initial infection by *Fusarium* pathogens (i.e. type I resistance). Thus, the antibody fusion conferred both type I and type II resistance in wheat plants. Moreover, some transgenic wheat lines display a higher level of type I resistance than Sumai3, the best natural FHB-resistant wheat cultivar available [4, 63, 68]. Analyses of yield components indicate that significantly more grains are produced in transgenic wheat than the nontransgenic controls [42].

We hypothesize a vital role played by the *Fusarium*-specific antibody in the enhanced resistance in transgenic plants. The antibody derived from chicken was isolated by phage display with the mycelium cell wall-bound proteins [16] from a highly virulent strain 5035 of *F. asiaticum* that was originally isolated from a scabby wheat spike in Wuhan, a frequent FHB epidemic region in China [41, 66]. Immunofluorescence localization studies reveal a clear, specific binding of the scFv antibody to the surface of the FHB pathogen, suggesting the presence of surface antigens on the *F. asiaticum* mycelium or infection structures. The antibody fusion shows a high binding specificity and affinity towards *Fusarium* species and strongly inhibits the fungal growth by damaging the mycelium cell walls *in vitro* [16]. It is likely that the *Fusarium*-specific antibody within the fusion protein actively targets the fusion into the fungal infection structures in plant tissues whereby the antibody directly interacts with the fungal antigens and the antifungal peptide damages the mycelium membranes to interfere the fungal development [16, 69, 70]. The combined two molecules can display synergistic, specific bi-functional activities towards the pathogens. Constitutive expression *in planta* regulated by the maize ubiquitin promoter would provide a continual supply of the antibody fusion proteins that are secreted into apoplast and/or accumulated surrounding the fungal infection structures in plant tissues, resulting in an effective restriction of the fungal infection and growth. Controlling fungal spreading and development on wheat spikes in field is a key step at fountainhead point to reduce or eliminate *Fusarium* mycotoxins in cereal grains. *Fusarium*-specific antibodies fused to antifungal peptides are ideal molecules for constitutive expression in cereal crops that grow in field.

## 5. Conclusions

Antibodies show high specificity and affinity towards antigens. Recombinant scFv antibodies and their corresponding genes can be isolated simultaneously by phage display. Antibodies generated against the mycelium surface antigens of *Fusarium* mycotoxin-producing fungi bind specifically to the fungal cell walls. scFv antibodies are genetically manipulated with ease to create scFv-AFP fusion proteins that display synergistic, bi-functional activity specific to *Fusarium* pathogens *in vitro* and *in planta*. Various forms of antibodies have been expressed in plants and plant-expressed antibodies are functionally similar to their counterparts that are derived from mammalian cells. Transgenic expression of antibodies protects plants from pathogens and a *Fusarium*-specific scFv antibody confers a significant enhancement of resistance to *Fusarium* pathogens. A more significantly enhanced resistance to *Fusarium* pathogens is obtained in transgenic plants expressing a *Fusarium*-specific antibody fused to an antifungal protein. *Fusarium*-specific antibody is essential for the enhanced resistance in plants. This is the first demonstration for fungal resistance by exploiting fungus-specific scFv antibody fusions and more efforts are required to obtain antibody-mediated resistance to other fungal pathogens. Methods for generation of transgenic cereal plants without any antibiotics or herbicide marker genes are established. Combined with the advances of plant biotechnology, antibody-mediated resistance to initial infection and spreading of *Fusarium* head blight pathogens on wheat spikes provides new opportunities for the development of environmentally friendly mycotoxin control strategies for cereals in field and during storage.
